# Editorial: A Novel Perspective for Photosystem I: An Emerging Hub for the Functional Integration of Photosynthesis and Metabolism

**DOI:** 10.3389/fpls.2022.871623

**Published:** 2022-04-12

**Authors:** Nicolás E. Blanco, Peter J. Gollan, Virginie Mengin, Lauri Nikkanen, Corina M. Fusari

**Affiliations:** ^1^Centre of Photosynthetic and Biochemical Studies (CEFOBI-CONICET-UNR), Rosario, Argentina; ^2^Molecular Plant Biology, Department of Life Technologies, University of Turku, Turku, Finland; ^3^Schools of Life Sciences, University of Essex, Colchester, United Kingdom

**Keywords:** photosystem I, photosynthesis, metabolism, environmental stress, electron transport

The autotrophic lifestyle of photosynthetic organisms is determined by their capacity to assimilate CO_2_ into organic molecules using sunlight as the source of energy. CO_2_ assimilation provides the bases to sustain all aspects of plant growth, including division, expansion, function and maintenance of cells, and production of stress and defense metabolites. Photosynthetic electron transport (PET) couples absorbed light energy to a chain of redox reactions, leading to formation of a proton motive force and generating NADPH and ATP that are required in the subsequent steps of CO_2_ fixation. Behind this definition of photosynthesis, there are multiple complex processes associated with the coordination of PET and CO_2_ assimilation (Stirbet et al., 2020). Energy conversion via photochemical reactions, and gas exchange that facilitates accessibility and conversion of CO_2_ to photoassimilates are mutually dependent and directly influenced by environmental conditions (Matuszynska et al., 2019; Walker et al., 2020). Adaptation of photosynthesis to external cues has been explored with a focus either on intrinsic aspects of the photochemical process (Alric and Johnson, 2017; Tikhonov and Vershubskii, 2017), or on the dynamic of CO_2_ assimilation via Rubisco carboxylation/oxygenation (Von Caemmerer, 2020). Likewise, primary metabolic responses to environmental factors have usually involved the characterization of enzymatic activities and central metabolites (e.g., Rubisco, sucrose, starch and protein biosynthetic pathways), independently of photosynthetic parameters (Stitt et al., 2010). Research on further metabolic processes, such as glycolysis, TCA cycle or lipid metabolism have also addressed the connection with photosynthesis mostly indirectly, as a downstream proxy of changes seen in photosynthetic yield.

This Research Topic offered an opportunity to present work investigating simultaneously photosynthesis and primary metabolism from a novel perspective. To encourage more integrative research, our proposal was designed to explore photosynthetic energy transduction and CO_2_ assimilation from photosystem I (PSI) point of view. Recent mounting evidence has demonstrated that PSI is a critical point in the photosynthetic machinery to respond against sudden changes in central metabolism and/or growing conditions. This is in part due to simultaneous regulatory roles in both upstream photochemical reactions, through the control of photo-protective mechanisms, and downstream metabolic pathways, including CO_2_ fixation and redox levels. Furthermore, the sensitivity of PSI to the imbalance between energy production and cell-metabolism demands, a phenomenon called PSI-acceptor side limitation, makes PSI an excellent gauge of non-optimal external conditions (Pollastrini et al., 2020). In this Research Topic, the reader can find a set of articles demonstrating the intimate connection between central metabolism and photosynthetic performance with focus on PSI.

The recent advances in the comprehension of the link between plastid fatty acid metabolism and redox status are presented in a mini-review by Hernández and Cejudo. Both the steps of the biosynthetic pathways for *de novo* fatty-acid production and the role of these molecules in photosynthetic performance and in response to abiotic stress are discussed as part of the chloroplast function. In addition, the authors identified key steps in lipid metabolism regulated by Thioredoxin (Trx)- NADPH-dependent Trx reductase C (NTRC) at the stroma.

The latest findings on the molecular mechanisms of PSI photoprotection are exposed by Krämer and Kunz. There, they focused on the role of the malate valve as a mechanism of dissipating reducing capacity outside the chloroplast. Their work spotlights not only the stromal NAD(H) as an underappreciated source to mitigate PSI damage, but also the activity of two malate dehydrogenases that explain the generation of NAD(H) in the stroma.

Modeling photosynthesis provides a framework to organize and test hypotheses about specific processes experimentally, revealing targets for engineering photosynthesis. Saadat et al. present an update of the previously published mechanistic model linking the PET chain with photosynthetic primary metabolism (Matuszynska et al., 2019). This implementation mostly focused on changing the description of the PSI mechanism from the original model by including reactive oxygen species (ROS) formation and metabolism. Interestingly, it highlighted a significant impact of Sedoheptulose-1, 7-bisphosphatase on the regulation of ROS scavenging mechanisms in saturated CO_2_ conditions.

Ogawa et al. monitored the kinetics of chlorophyll and NADPH fluorescence during photosynthetic induction in order to elucidate the dynamics between photosynthetic and respiratory electron transport in the model cyanobacterium *Synechocystis* sp. PCC 6803. In cyanobacteria, both photosynthetic and respiratory pathways occur on the same thylakoid membranes and share common components such the plastoquinone pool, which necessitates strict coordination of different pathways in order to maintain redox balance in variable environmental conditions. By using mutants deficient in respiratory/cyclic electron transport or the oxidative pentose phosphate pathway, and by studying the effect of glucose as an exogenous electron source, the authors identified the redox state of the cellular NADPH/NADP^+^ pool as a key redox sensor at the intersection of photosynthetic and respiratory pathways.

The research by Shimakawa et al. investigated distinct pathways of electron donation from PSI to O_2_ via photorespiration or flavodiiron (FLV) protein activity. Both strategies achieve oxidation of the P700 reaction center, but are differently utilized during the evolution of photosynthesis. The authors used photosynthetic measurements under standard and low O_2_ treatments, in wild type and FLV-deficient strains of the bryophyte *Marchantia polymorpha*, to show that, while photorespiration is active in *M. polymorpha*, FLV has a dominant role in P700 oxidation at atmospheric CO_2_.

Finally, Lima-Melo et al. summarized the physiological function of PSI including its main role as part of PET. Special attention is placed on the onset of PSI photoinhibition, the multiple mechanisms participating in PSI protection and the link to stromal sink capacity. Furthermore, ROS production and associated retrograde signaling pathways are introduced as part of the response to changes in the equilibrium between PSI inactivation and activity. The authors also introduce a set of open questions about PSI and provide hints about the direction of the research in this topic.

At the light of the positive feedback that we received in this first volume of our Research Topic, we are happy to announce the release of a follow-up issue. With this initiative, we hope to broaden the scope by reaching out for more studies integrating aspects of photosynthesis and metabolism. Please find more info at: https://www.frontiersin.org/research-topics/31418/the-back-and-forth-between-metabolism-and-photosystem-i-volume-ii.

We are looking forward to your contributions that drive further understanding of the rich and complex operation of photosynthetic organisms.

## Author Contributions

All authors listed have made a substantial, direct, and intellectual contribution to the work and approved it for publication.

## Conflict of Interest

The authors declare that the research was conducted in the absence of any commercial or financial relationships that could be construed as a potential conflict of interest.

## Publisher's Note

All claims expressed in this article are solely those of the authors and do not necessarily represent those of their affiliated organizations, or those of the publisher, the editors and the reviewers. Any product that may be evaluated in this article, or claim that may be made by its manufacturer, is not guaranteed or endorsed by the publisher.
